# Teachers’ Self-Efficacy: The Role of Personal Values and Motivations for Teaching

**DOI:** 10.3389/fpsyg.2019.01645

**Published:** 2019-07-12

**Authors:** Daniela Barni, Francesca Danioni, Paula Benevene

**Affiliations:** ^1^Department of Human Sciences, LUMSA University, Rome, Italy; ^2^Family Studies and Research University Centre, Catholic University of Milan, Milan, Italy

**Keywords:** teachers, self-efficacy, values, motivations for teaching, well-being

## Abstract

Teachers’ personal values drive their goals and behaviors at school. Moreover, values can support subjective well-being and an individual sense of self-efficacy. Teachers’ self-efficacy, namely teachers’ beliefs in their ability to effectively handle the tasks, obligations, and challenges related to their professional activity, plays a key role in influencing important academic outcomes (e.g., students’ achievement and motivation) and well-being in the working environment. Based on Schwartz’s well-known theory of human values, this study sought to examine the relations between teachers’ values (i.e., conservation, openness to change, self-transcendence, and self-enhancement) and their self-efficacy. In particular, it aimed at analyzing the extent to which these relations are moderated by teachers’ controlled and autonomous motivations for teaching. Two hundred and twenty-seven Italian high school teachers (73.6% females; *M* = 44.77 years, *SD* = 10.56) were involved in the study and asked to complete a self-report questionnaire. Results showed that teachers’ conservation values were positively associated to sense of self-efficacy regardless of the type and level of motivation for teaching. More interestingly, the relationships between openness to change and self-efficacy on the one hand, and self-transcendence and self-efficacy on the other, varied depending on teachers’ motivations. These relations were stronger when teachers perceived less external pressure and felt to be self-determined toward teaching. Implications of these results for teachers’ practices and well-being in their work environment and further developments of the study are discussed.

## Introduction

Teachers’ self-efficacy has progressively gained an important role in school psychology research as a result of its implications for teaching effectiveness, instructional practices, and for students’ academic achievement (Klassen et al., 2009; Klassen and Tze, 2014). Considerable research has shown that teachers with high levels of self-efficacy experience higher levels of job satisfaction, lower levels of job-related stress and face less difficulties in dealing with students’ misbehaviors (Caprara et al., 2003). Thus, understanding the main antecedents of self-efficacy may have important payoffs in working for teachers’ well-being and school effectiveness and improvement.

The concept of self-efficacy derives from Bandura’s social-cognitive theory of behavioral change (Bandura, 1977). It refers to a teacher’s belief in his/her ability to successfully cope with tasks, obligations and challenges related to his/her professional role (e.g., didactical tasks, managing discipline problems in the class, etc.) (Caprara et al., 2006). This belief is determined by several factors, among which personality characteristics – in particular personality traits – which have led to a rise in the academic interest on the topic. For example, based on the five-factor model of personality (Costa and McCrae, 1992), Djigić et al. (2014) found that teachers with higher levels of openness to experience and conscientiousness reported a stronger sense of efficacy. Sousa et al. (2012), in their study involving frontline service employees, showed that also personal values are significant predictors of workers’ self-efficacy. Specifically, openness to change values (i.e., self-direction, stimulation, and hedonism) and self-enhancement values (i.e., power and achievement) are both positively related to self-efficacy. These values are part of the well-known Schwartz’s (1992) model of human values, which also includes conservation values (i.e., tradition, conformity, and security) and self-transcendence values (i.e., benevolence and universalism). According to Schwartz, personal values can be defined as *trans*-situational goals that vary in importance and serve as guiding principles in people’s lives. Schwartz (1992, 2012) identified ten basic value types: power (social status, dominance over people and resources), achievement (personal success according to social standards), hedonism (pleasure or sensuous gratification), stimulation (excitement, challenge, and novelty), self-direction (independence of thought and action), benevolence (preserving and enhancing the welfare of people to whom one is close), universalism (understanding, tolerance, and concern for the welfare of all people and nature), tradition (respect and commitment to cultural or religious customs and ideas), conformity (restraint of actions and impulses that may harm others or violate social expectations), and security (safety and stability of society, relationships, and self). These ten values can be organized into a motivational two-dimensional system. The first dimension contrasts openness to change, which emphasizes change and independence, and conservation, where the emphasis is instead on self-restraint, preserving traditional practices, and safeguarding stability. The second dimension contrasts self-enhancement, where people prioritize their personal interests even at the expense of others, and self-transcendence, where people transcend their selfish concerns to promote the welfare of others. More recently, Schwartz (2012) has highlighted that self-transcendence and conservation values are characterized by a social focus, that is concern with outcomes for others or for established institutions, whereas self-enhancement and openness to change values by a personal focus, namely concern with outcome for self.

Despite their great influence on many attitudes and behaviors (Schwartz, 2015), also in the workplace (e.g., Koivula, 2008) and at school (e.g., Barni et al., 2018), the relationship between personal values and self-efficacy has been completely under investigated among teachers. This study intends to fill this gap. In particular, we analyzed in a sample of 227 teachers how a comprehensive set of personal values (i.e., conservation, openness to change, self-transcendence, and self-enhancement) is related to self-efficacy, and whether this relationship is moderated by controlled versus autonomous motivations for teaching. According to the Self-Determination Theory (Deci and Ryan, 1985), control-motivated teachers perform teaching activities because they wish to receive external rewards (e.g., school principal’s approval) or to avoid feelings of guilt, whereas autonomously motivated teachers perform teaching activity because of the intrinsic value they attribute to it (Roth et al., 2007). Given that values to exert influence must be activated and the target behavior needs to be personally relevant to the individual (Bardi and Schwartz, 2003), it is likely that the relation between values and self-efficacy is stronger when teaching behavior is self-determined (i.e., low controlled motivations and high autonomous motivations). Indeed, as typically shown by studies dealing with the person-situation controversy, the stronger the perceived situational pressure to hold a value or to act in a particular way, the weaker should be the importance of stable individual differences such as personal values (e.g., Bardi and Schwartz, 2003; Barni et al., 2016; Danioni and Barni, 2017).

## Method

### Participants and Procedure

Participants were 227 teachers (73.6% females) working in Italian high schools whose age ranged between 25 and 68 years (*M* = 44.77, *SD* = 10.56). On average, the teachers involved had been working in the schools for 7.93 years (*SD* = 7.80, range = 1–34). Most of the participants were tenured teachers (95.1%); 43.3% of them taught scientific and technical subjects, 41.0% taught humanistic subjects whereas the remaining 15.7% taught foreign languages. Participants were recruited with the collaboration of the schools where they worked and were informed about the main objectives of the study. The researcher directly contacted the school principal to obtain approval to carry out the research, who then introduced the researcher to the teachers. Teachers participated in the study on a voluntary basis. Overall, sixteen schools were involved.

Teachers who gave written informed consent were informed about the main objectives of the study and filled in an anonymous self-report questionnaire (response rate: 72.7%). The study was approved by the Ethics Committee of the Catholic University of Milan and followed the APA ethical guidelines of research. Data treatment follows the Italian legal and privacy restrictions (Italian Data Protection Code – Legislative Decree No. 196/2003) and the General Data Protection Regulation 2016/679.

### Measures

#### Personal Values

The Portrait Values Questionnaire (PVQ) (Schwartz et al., 2001; Italian validation by Capanna et al., 2005) was used to measure personal values. The PVQ is composed of 40 verbal portraits of a person and his/her objectives or aspirations, which indirectly reflect the importance of a value. For example, “Thinking up new ideas and being creative is important to him/her. He/She likes to do things in his/her own original way” describes a person for whom openness to change is important. Respondents’ values are inferred from their self-reported similarity (from 1 = not like me at all to 6 = very much like me) to people described. We computed four mean indexes assessing the importance personally given to conservation (α = 0.86), openness to change (α = 0.79), self-transcendence (α = 0.80), and self-enhancement (α = 0.77).

#### Motivations for Teaching

The Autonomous Motivations for Teaching Scale (Roth et al., 2007), translated into Italian and back-translated into English, was used to assess motivations for teaching. This scale includes 16 items on a 5-point Likert answer scale (from 1 = totally disagree to 5 = totally agree). Eight items measure teachers’ controlled motivations for teaching (e.g., “When I devote time to individual talks with students, I do so because I want the parents to appreciate my knowledge and familiarity with their children;” α = 0.74) and the remaining eight items measure teachers’ autonomous motivations for teaching (e.g., “When I devote time to individual talks with students, I do so because I like being in touch with children and adolescents;” α = 0.65).

#### Self-Efficacy

The Teachers’ Self-efficacy Scale (Caprara, 2001) was used. The scale is composed of 12 items on a 7-point Likert answer scale (from 1 = totally disagree to 7 = totally agree). An example of an item is: “As a teacher, I am capable of getting recognition and appreciation from my students”. We computed one mean index measuring teachers’ beliefs in their ability to effectively handle various tasks, obligations, and challenges related to their professional role (α = 0.83).

### Data Analysis

#### Preliminary Analysis

We described the study variables in terms of means, standard deviations, and range. Associations between variables were measured by bivariate Pearson correlations.

#### Moderation of Controlled and Autonomous Motivations for Teaching on the Relation Between Teachers’ Personal Values and Self-Efficacy

We conducted two multiple hierarchical regressions to test the moderation hypothesis. Teachers’ personal values (i.e., conservation, openness to change, self-transcendence, and self-enhancement) were used as independent variables, testing separately the interaction effects with the two moderators (i.e., controlled motivations and autonomous motivations for teaching) for the outcome of teachers’ self-efficacy.

Each of the two regression models, the first one with teachers’ controlled motivations as moderator and the second one with autonomous motivations as moderator, was tested in the following steps. In step 1 predictors (i.e., teachers’ personal values) and moderator variable (i.e., teachers’ controlled motivations or autonomous motivations) were entered in the model to test their direct relations with teachers’ self-efficacy. In step 2 the interaction term between predictor and moderator variable was entered to the model to test the statistically significance of the interaction effect. The independent variables were centered on their means before computing the interaction terms to minimize multicollinearity and for easier interpretation of model coefficients (Aiken and West, 1991). Lastly, simple slope analysis was performed to probe any significant interaction effect. We used SPSS 24.0 to conduct all the analyses.

## Results

Descriptive statistics of the study variables and their intercorrelations are reported in Table 1. Teachers gave the greatest importance to self-transcendence values, followed by conservation, openness to change, and self-enhancement. They scored higher on autonomous motivations for teaching than on controlled motivations, *t*(225) = 25.22, *p* < 0.001, and on average they reported a high level of self-efficacy.

**Table 1:** Pearson correlation coefficients, means, standard deviations (*SD*) and ranges of teachers’ values, motivations for teaching, and self-efficacy (*N* = 227).

	1	2	3	4	5	6	7
(1) Conservation	1	0.20^**^	0.37^**^	0.22^**^	0.12	0.13	0.28^**^
(2) Openness to change		1	0.27^**^	0.55^**^	0.03	0.17^**^	0.19^**^
(3) Self-transcendence			1	0.04	-0.17^**^	0.20^**^	0.22^**^
(4) Self-enhancement				1	0.14^*^	0.15^*^	0.07
(5) Controlled motivations					1	0.48^**^	-0.01
(6) Autonomous motivations						1	0.11
(7) Self-efficacy							1
Mean	3.86	3.82	4.86	2.79	2.98	3.90	5.37
*SD*	0.82	0.74	0.63	0.96	0.57	0.49	0.68
Range	2–6	2–6	3–6	1–6	1–5	2–5	3–7

*^∗∗^p < 0.01 and ^∗^p < 0.05.*

Teachers’ personal values were all significantly correlated to each other, with the only exception of self-transcendence and self-enhancement values. Openness to change, self-transcendence, and self-enhancement were positively associated with teachers’ autonomous motivations for teaching. Self-enhancement and self-transcendence were also associated, respectively positively and negatively, with teachers’ controlled motivations. Finally, teachers’ self-efficacy was significantly correlated to conservation, openness to change and self-transcendence, but not to self-enhancement values or motivations for teaching (Table 1).

**FIGURE 1:**
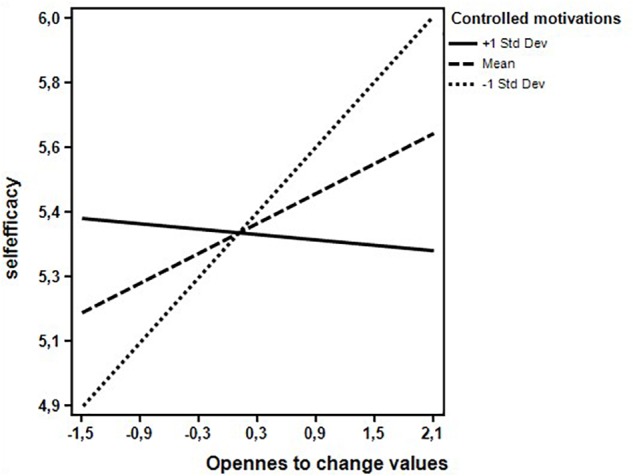
Interaction effect between teachers’ openness to change values and controlled motivations on self-efficacy.

**FIGURE 2:**
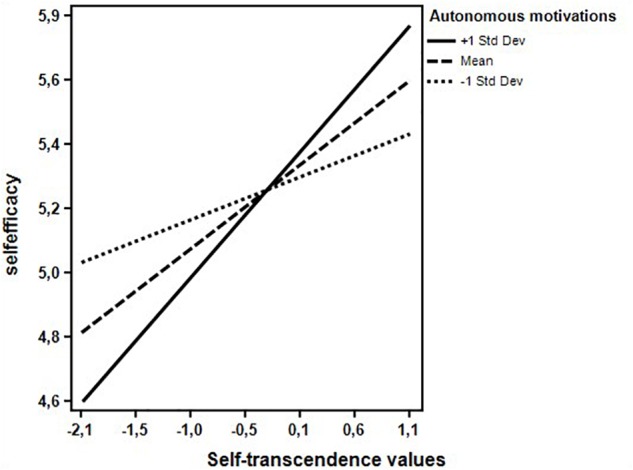
Interaction effect between teachers’ self-transcendence values and autonomous motivations on self-efficacy.

Table 2 shows the results of moderated regression analyses. Both models, the first one (Model 1) with controlled motivations as moderator and the second (Model 2) with autonomous motivations as moderator, were statistically significant [Model 1: *F*(9,215) = 4.063, *p* < 0.001; Model 2: *F*(9,215) = 3.391, *p* < 0.01]. They both accounted for significant amount of variance in teachers’ self-efficacy (Model 1: *R*^2^ = 0.145, *p* < 0.001; Model 2: *R*^2^ = 0.135, *p* < 0.001). Teachers’ conservation values were positively associated to self-efficacy regardless of the type and level of motivations for teaching. The relations of openness to change and self-transcendence with the criterion variable were instead moderated by teachers’ motivations. Examination of the interaction plot showed that at low levels of controlled motivations (1 standard deviation below the mean), teachers’ openness to change supported their sense of efficacy (Figure 1). At higher levels of autonomous motivations (1 standard deviation above the mean), a positive association between self-transcendence and self-efficacy emerged and was stronger than that found at low levels of autonomous motivations (Figure 2). Finally, teachers’ self-enhancement values were not related with self-efficacy.

**Table 2:** Moderated regression analysis results (dependent variable: teachers’ self-efficacy).

Variables		Model 1			Variables		Model 2		
Step 1	β	*t*	*p*	*R*^2^	Step 1	β	*t*	*p*	*R*^2^
				0.104^∗∗^					0.107^∗∗^
Conservation	0.230	3.217	0.001		Conservation	0.224	3.185	0.002	
Openness to change	0.134	1.677	0.095		Openness to change	0.130	1.634	0.104	
Self-transcendence	0.094	1.275	0.204		Self-transcendence	0.091	1.253	0.212	
Self-enhancement	-0.056	-0.711	0.478		Self-enhancement	-0.064	-0.812	0.418	
Controlled motivations (CM)	-0.022	-0.336	0.737		Autonomous motivations (AM)	0.053	0.799	0.425	

**Step 2**	**β**	***t***	***p***	**Δ*R*^2^**	**Step 2**	**β**	***t***	***p***	**ΔR^2^**

				0.041^∗^					0.028^∗^
Conservation × CM	0.018	0.227	0.821		Conservation × AM	-0.046	-0.628	0.531	
Openness to change × CM	-0.261	-2.991	0.003		Openness to change × AM	-0.070	-0.840	0.402	
Self-transcendence × CM	-0.043	-0.598	0.551		Self-transcendence × AM	0.149	1.949	0.053	
Self-enhancement × CM	0.138	1.591	0.113		Self-enhancement × AM	0.004	0.044	0.965	

*^∗∗^p < 0.01 and ^∗^p < 0.05. CM, controlled motivations; AM, autonomous motivations.*

## Discussion

Teachers’ self-efficacy has been repeatedly demonstrated to be a relevant factor for the effectiveness of the teaching activity, as it is a powerful drive influencing the behavior of teachers in the classroom and the effort put in the endeavor (Klassen et al., 2009; Klassen and Tze, 2014). Therefore, improved teacher self-efficacy can result in improved teacher mental health and job satisfaction, and students’ academic performance (Bandura, 1977).

Based on this, it becomes of extreme relevance to understand what influences teacher’s belief in his/her ability to successfully cope with tasks, obligations and challenges related to his/her professional role (Caprara et al., 2006; Tschannen-Moran and Hoy, 2007). With this purpose, teachers’ personal values may be something of great interest to explore. Indeed, values have been shown to effectively predict workers’ self-efficacy (e.g., Sousa et al., 2012), but despite the relevant heuristic power that personal values have in many life contexts, they have been completely under investigated in the school context. Besides trying to fill this gap of knowledge, the present study also aims at investigating whether and the extent to which teachers’ values–self-efficacy relation changes as a function of teachers’ motivations for teaching (Roth et al., 2007). Specifically, the relations between personal values and self-efficacy was expected to be stronger for self-determined teachers, namely those teachers characterized by low controlled motivations (i.e., performing teaching activity because of external rewards) and high autonomous ones (i.e., attributing an intrinsic value to their professional activity). Self-determination may activate values and related behaviors, thus strengthening the values–self-efficacy relation.

From this study it emerged that teachers gave the greatest importance to self-transcendence and conservation values. This result is in line with previous studies on teachers (e.g., Verkasalo et al., 1996) but also with studies on the Italian adult population in general (e.g., Caprara et al., 2011). Thus, the teachers who participated in the study recognized the importance of values like the welfare of ingroup members, tolerance, social justice, world beauty (i.e., benevolence and universalism, conformity, security, and tradition). Interestingly, according to Schwartz’s (2012) refined model of values, self-transcendence and conservation are both values focused on social outcomes, namely values primarily regulating how one socially relates to others and affects them, whereas openness to change and self-enhancement are mostly focused on self-interest. Thus, in their value priorities teachers appear to be socially oriented, both in terms of caring for others (i.e., benevolence and universalism) and in terms of maintenance of the current order (i.e., tradition, conformity, and security).

All in all, teachers’ personal values were shown to be important predictors of teachers’ self-efficacy. Specifically, conservation was positively associated to teachers’ self-efficacy, both for those teachers driven by controlled motivations in their work and for those teachers driven by autonomous motivations. It is likely that respecting norms and safeguarding stability in the field of education (where teachers are supposed to feel the responsibility to transmit knowledge and to “take care” of students) makes teachers experience a sense of security in planning, organizing, and carrying out activities required to attain educational goals. The specific context where the study was carried out explains therefore the contrasting finding compared to the results by Sousa et al. (2012), where conservation had a negative impact on self-efficacy. The result is, however, more in line with Djigić et al. (2014), who found teachers’ conscientiousness to be positively related with self-efficacy.

The relations between openness to change and self-transcendence values and self-efficacy varied instead as a function of teachers’ motivations for teaching. Indeed, the relation between openness to change and self-efficacy was positive at low levels of controlled motivations, but negative at high levels of controlled motivations. In other words, teachers for whom values such as novelty, freedom, and choosing one’s own goals are important for their perception of efficiency actually feel more efficient in their teaching activities when they feel independent from external conditions. On the contrary, where the external pressures to behave in a certain way are strong, the more teachers give importance to openness to change, the more they perceive themselves as inefficient. According to the person-environment fit theory (see Caplan, 1987), this result shows that teachers’ self-efficacy is higher when their personal values and work environment attributes are perceived as compatible.

In this line, also a genuine interest for others – in terms of importance assigned to self-transcendence values – for teachers who recognized the intrinsic value of their teaching activity was found to be a significant factor in fostering teachers’ self-efficacy. Teachers who show concern for their students and “value” themselves in terms of what they do, can profit in terms of self-efficacy.

In interpreting these results, some limitations need to be taken into consideration. The main shortcoming of the present study is the involvement of a single-country convenience sample, since participants were selected according to the high school collaboration, and its cross-sectional design, which limits casual inferences from the data as well as possible considerations with regards to bidirectionality of the links. Caution is needed in generalizing results since the sample was mainly composed of female teachers (73.6%); however, this reflects the Italian reality, where the majority of teachers are women.

Based on the relevance of self-efficacy for teaching effectiveness and students’ academic achievement, future research may also address the role of other variables in influencing teachers’ self-efficacy. For example, it would be of great interest to expand the study of teachers’ self-efficacy by considering their emotional intelligence (Magnano et al., 2017b) and organizational mindfulness (Magnano et al., 2017a).

However, it is worth noting that, to the best of our knowledge, this is the first study focused on the relations between teachers’ personal values and their self-efficacy. We believe the strongest point of this study is to have shown that values, under certain motivational conditions, are precious resources to improve the quality of teaching experience for teachers and indirectly for their students. Self-knowledge and conscious endorsement of personal values could therefore help teachers to healthily handle the challenges of teaching and to prevent work-related stress and burnout. Further research about teachers’ values and their implications for teachers’ wellbeing and teaching practices should be promoted, particularly in culturally specific contexts.

## Data Availability

The datasets for this manuscript are not publicly available because of local legal and privacy restrictions (Italian Data Protection Code – Legislative Decree No. 196/2003). However, the raw data supporting the conclusions of this manuscript can be made available by the first author to qualified researchers upon request.

## Ethics Statement

The authors assert that all procedures contributing to this work comply with the ethical standards of the relevant national and institutional committees on human experimentation. All participants gave written informed consent in accordance with the Declaration of Helsinki. The study was approved by the Ethics Committee of the Catholic University of Milan, Italy.

## Author Contributions

DB designed and carried out the study, contributed to the analysis of the results and to the writing of the manuscript. FD contributed to the analysis of the results and to the writing of the manuscript. PB contributed to the writing of the manuscript.

## Conflict of Interest Statement

The authors declare that the research was conducted in the absence of any commercial or financial relationships that could be construed as a potential conflict of interest.
